# Steroidogenic factor-1 (SF-1, *NR5A1*) and human disease

**DOI:** 10.1016/j.mce.2010.11.006

**Published:** 2011-04-10

**Authors:** Bruno Ferraz-de-Souza, Lin Lin, John C. Achermann

**Affiliations:** Developmental Endocrinology Research Group, Clinical & Molecular Genetics Unit, UCL Institute of Child Health, University College London, London WC1N 1EH, United Kingdom

**Keywords:** ACT, adrenocortical tumor, AMH, anti-Müllerian hormone (also known as Müllerian-inhibiting substance), DBD, DNA-binding domain, DSD, disorders of sex development, LBD, ligand-binding domain, POI, primary ovarian insufficiency, Steroidogenic factor-1 (SF-1), NR5A1, Adrenal failure, 46,XY disorders of sex development (DSD), Primary ovarian insufficiency (POI), Infertility, Adrenocortical tumor, Endometriosis

## Abstract

Steroidogenic factor-1 (SF-1, Ad4BP, encoded by *NR5A1*) is a key regulator of adrenal and reproductive development and function. Based upon the features found in *Nr5a1* null mice, initial attempts to identify SF-1 changes in humans focused on those rare individuals with primary adrenal failure, a 46,XY karyotype, complete gonadal dysgenesis and Müllerian structures. Although alterations affecting DNA-binding of SF-1 were found in two such cases, disruption of SF-1 is not commonly found in patients with adrenal failure. In contrast, it is emerging that variations in SF-1 can be found in association with a range of human reproductive phenotypes such as 46,XY disorders of sex development (DSD), hypospadias, anorchia, male factor infertility, or primary ovarian insufficiency in women. Overexpression or overactivity of SF-1 is also reported in some adrenal tumors or endometriosis. Therefore, the clinical spectrum of phenotypes associated with variations in SF-1 is expanding and the importance of this nuclear receptor in human endocrine disease is now firmly established.

## 1 Introduction

Steroidogenic factor-1 (SF-1, *NR5A1*, Ad4BP) was originally identified as a master-regulator of steroidogenic enzymes in the early 1990s following the seminal work of Keith L. Parker and Ken-ichirou Morohashi (Rice et al., 1991; Lala et al., 1992; Morohashi et al., 1992). SF-1 has since been shown to control many aspects of adrenal and reproductive function, and many factors involved in the development of these structures have been shown to be regulated by SF-1 (Parker and Schimmer, 1997; Lin and Achermann, 2008; Schimmer and White, 2010).

In addition to numerous *in vitro* studies, significant early progress in understanding the *in vivo* function of SF-1 was obtained following deletion of the gene encoding Sf-1 (*Nr5a1*) in the mouse (Luo et al., 1994; Sadovsky et al., 1995; Shinoda et al., 1995). Homozygous null mice (−/−) have adrenal agenesis, complete testicular dysgenesis, persistent Müllerian structures in XY animals, partial hypogonadotropic hypogonadism, and other features such as hyposplenism, abnormalities of the ventro-medial hypothalamus and late-onset obesity (Majdic et al., 2002). Haploinsufficient animals were originally thought to have minimal phenotypes compared to the severe features seen in *Nr5a1* null littermates. However, more subtle phenotypes have also been found to be present in haploinsufficient animals following more detailed investigation (Bland et al., 2000, 2004), and newer molecular and transgenic strategies continue to elucidate the role SF-1 plays as a critical mediator of endocrine development and function (Ferraz-de-Souza et al., 2009; Hoivik et al., 2010).

In parallel with these *in vitro* and *in vivo* studies, it has emerged that SF-1 is also an important factor in several human diseases. It is more than a decade since the first case of human SF-1 disruption was described. At that time it seemed likely that SF-1 changes in humans would be rare events associated with specific phenotypes. However, more recent studies are revealing that variations in SF-1 may play a much greater role in human disease than was originally thought. Here, we review the range of human phenotypes that are emerging in association with SF-1/*NR5A1* variants. It is also worth noting that – to date – no complete deletions of *NR5A1* have been described; thus, it is not completely established whether complete loss of SF-1 is compatible with embryonic or fetal survival in humans.

## 2 The “classic” phenotype: adrenal and gonadal failure

Initial attempts to find *NR5A1* mutations in humans focussed on individuals with primary adrenal insufficiency and complete 46,XY gonadal dysgenesis, a phenotype resembling the *Nr5a1* knock-out mouse. This phenotype is very rare in humans, but *NR5A1* mutations were identified and reported in two 46,XY patients with female genitalia, Müllerian structures (uterus and upper vagina), complete gonadal dysgenesis and primary adrenal failure.

**Fig. 1:**
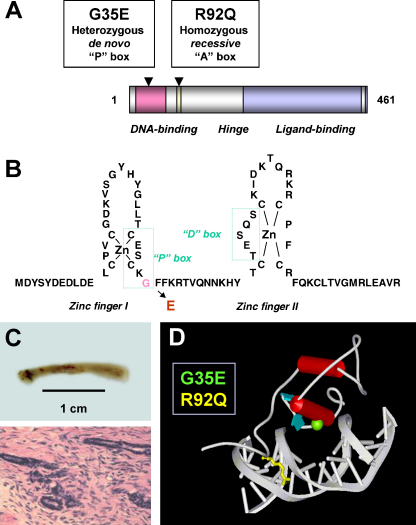
Overview of SF-1 mutations associated with adrenal failure and 46,XY DSD. (A) Cartoon of SF-1 showing key functional domains and the position of the two SF-1 variants associated with this phenotype. (B) The amino-acid structure of the zinc fingers of the DNA-binding domain of SF-1 showing the proximal (P)-box motif and the glycine residue that is altered by the p.G35E mutation. (C) Streak-like gonads removed from the patient with the p.G35E change in late childhood (upper panel), containing poorly formed seminiferous tubules and connective tissue (lower panel). (D) Model of the DNA-binding domain of SF-1 bound to DNA based on the crystal structure of NGFI-B. The glycine at amino acid 35 (green) of the P-box forms the primary DNA-binding interface with the major groove of DNA and heterozygous disruption of this residue with replacement by glutamic acid is associated with a severe phenotype. In contrast, the arginine at amino acid 92 (yellow) affects the A-box of SF-1, which is important in stabilizing nuclear receptor binding. In this situation, homozygous disruption of the residue was associated with the severe adrenogonadal phenotype. Panels B and C reproduced with permission from Achermann et al. (1999) (copyright: Nature Publishing Group 1999). Panel D reproduced with permission from Achermann et al. (2002) (copyright: The Endocrine Society 2002).

The first individual described with this phenotype was found to have a *de novo* heterozygous p.G35E change in the P-box of SF-1 (Fig. 1A and B) (Achermann et al., 1999). This patient presented with salt-losing adrenal failure in early infancy and was thought to have a high block in steroidogenesis affecting both adrenal and testicular function (e.g. CYP11A1, STAR). However, the identification of a streak-like gonad (Fig. 1C) and Müllerian structures was consistent with testicular dysgenesis rather than a steroidogenic defect, so disruption of a common developmental regulator such as SF-1 was hypothesized.

The p.G35E change affects a region that is critical for SF-1 function. It had been known for some time that the P-box of nuclear receptors is important in dictating DNA binding specificity through its interaction with DNA response elements in the regulatory regions of target genes (Umesono and Evans, 1989). SF-1 is thought to bind to estrogen receptor-like response elements as a monomer. The P-box motif forms the principle DNA-binding interface of SF-1 by interacting with the major groove of DNA (Fig. 1D). *In vitro* studies showed variable effects of this p.G35E alteration on DNA-binding and gene transactivation but with a significant loss of function in most cases (Ito et al., 2000). Although no strong dominant negative effects were seen, the p.G35E change may have a mild competitive or dominant negative effect in certain assay systems, or through interaction with co-regulators (Ito et al., 2000; Tremblay and Viger, 2003). Therefore, the phenotype may be the result of reduced SF-1 transactivation on multiple targets throughout the genome and at different stages of development. It is also not known whether skewed allelic expression could have resulted in different effects of this *NR5A1* mutation in different organs or whether – despite extensive analysis – a convert regulatory or intronic change could have reduced transcription or translation of the wild-type allele. Nevertheless, this case established that disruption of SF-1 could be associated with severe gonadal defects and significant adrenal dysfunction in humans.

The importance of SF-1 in regulating human adrenal and gonad development and function was confirmed following the report of an infant with a similar phenotype (primary salt-losing adrenal failure, 46,XY DSD, Müllerian structures) who had inherited a homozygous p.R92Q alteration in SF-1 in a recessive fashion (Fig. 1A) (Achermann et al., 2002). This change lies within the A-box of SF-1 and interferes with monomeric DNA binding stability (Fig. 1D). Again, the effects of this homozygous change are complex and variable, but in a range of promoter assays mean functional activity was in the order of 30–40% of wild-type (Ito et al., 2000; Achermann et al., 2002; Lin et al., 2007). However, the question of altered allelic expression or other genomic events was not an issue as the mutation was present on both alleles. The limited data available showed normal adrenal function in heterozygous carriers of this change, suggesting that disruption of both alleles is needed for the phenotype to be seen.

## 3 SF-1 and adrenal insufficiency

Given the importance of SF-1 in adrenal development, the next obvious question to address was whether SF-1 changes could be detected in girls (46,XX) who present with primary adrenal insufficiency or in patients with adrenal insufficiency where no specific cause had been found.

### 3.1 46,XX primary adrenal failure

**Fig. 2:**
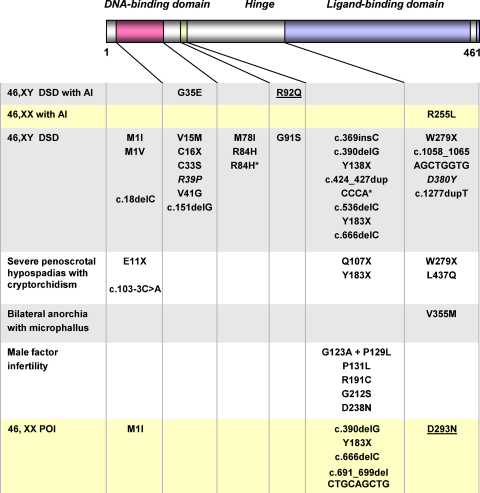
Overview of reported changes in SF-1/*NR5A1* in humans. The approximate location of the change within the predicted structure of SF-1 is shown. Yellow shaded areas represent changes found in 46,XX girls or women. Grey and white areas represent changes in individuals with a 46,XY karyotype. Underlined changes were detected in a homozygous state. All other changes were present in a heterozygous state. An asterisk denotes cases where the p.G146A polymorphism was also detected. The missense mutation shown in italics is predicted to disrupt function, but data from functional assays have not been reported. In addition, deletion of *NR5A1* on one allele has been reported as part of a contiguous gene deletion syndrome. AI, adrenal insufficiency; DSD, disorder of sex development, POI, primary ovarian insufficiency. Modified with permission from Lin et al. (2007) (copyright: The Endocrine Society 2002). For a complete overview of clinical and biochemical features associated with these changes, see Köhler & Achermann (2010).

In 2000, Biason-Lauber and Schoenle described a *de novo* heterozygous *NR5A1* change in a girl who had presented at 14 months of age with primary adrenal insufficiency and seizures (Fig. 2) (Biason-Lauber and Schoenle, 2000). The nucleotide transversion found was predicted to result in a p.R255L mutation in the proximal part of the ligand-like binding domain of the protein. The mutant SF-1 protein was reported to be transcriptionally inactive, but without a dominant negative effect. Of note, ovaries were detected by MRI scan and inhibin A was low normal for age, suggesting that this change in SF-1 had not disrupted ovarian development and that early indicators of ovarian function were intact. Thus, it appeared that SF-1 could be a cause of primary adrenal insufficiency in 46,XX girls.

### 3.2 Adrenal insufficiency of unknown etiology

The association of SF-1/*NR5A1* mutations with adrenal dysfunction raised the question whether specific changes in SF-1 could be found in patients with primary adrenal failure but in whom gonadal function appeared normal or only minimally affected. As SF-1 might interact with different co-factors and other co-regulators in different developing tissues, it was hypothesized that phenotypes affecting predominantly the adrenal gland or predominantly the gonad might exist. To address this, mutational analysis of *NR5A1* was undertaken in a cohort or phenotypic males (46,XY) and females (46,XX) with primary adrenal failure of unknown etiology, as well as a small group of 46,XY individuals with primary adrenal insufficiency and varying degrees of testicular dysfunction (Lin et al., 2006). Although changes in the related nuclear receptor DAX-1/*NR0B1* were found in a substantial proportion of boys with primary adrenal hypoplasia, no additional SF-1/*NR5A1* mutations were found that could be responsible for a predominantly adrenal phenotype. It seems, therefore, that changes in SF-1 are a relatively rare cause of primary adrenal failure in humans and that any such alterations might be expected to be associated with significant underandrogenization if present in 46,XY individuals.

## 4 SF-1 and 46,XY DSD

In contrast to the relatively small number of *NR5A1* changes reported in association with adrenal failure, heterozygous *NR5A1* changes are emerging as a relatively frequent finding in patients with 46,XY disorders of sex development (46,XY DSD) but *without* adrenal insufficiency (Fig. 2) (Lin and Achermann, 2008; Köhler et al., 2009). The first report of a heterozygous frameshift mutation resulting in disruption of SF-1 appeared in 2004 in a 46,XY female from Brazil who had presented with hypertension in early adulthood and who was found to have ambiguous genitalia (Correa et al., 2004). No gonadal tissue could be identified at laparoscopy. Two further case reports of heterozygous changes that are predicted to produce nonsense or frameshift changes in *NR5A1* appeared soon after in patients from Japan and France, respectively (Hasegawa et al., 2004; Mallet et al., 2004). As adrenal function was normal in all these cases, it was proposed that genetic events leading to haploinsufficiency of *NR5A1* could disrupt testicular development and function, whilst adrenal function – at least until early adulthood – remained intact. Two of these cases had arisen spontaneously and were likely to be *de novo* events, whereas parental DNA was not available in the third case.

### 4.1 The typical 46,XY DSD phenotype

**Fig. 3:**
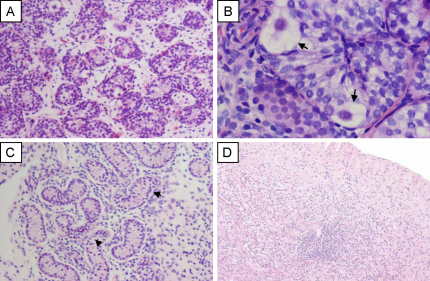
Gonadal histology in 46,XY and 46,XX patients with *NR5A1* mutations. (A and B) Partial testicular dysgenesis in a patient with 46,XY DSD without adrenal failure. Testicular architecture was largely intact but seminiferous tubules were reduced in size. Sertoli, germ and Leydig cells were present. (original magnification 100× and 400× respectively; germ cells shown by arrows). (C) Testicular biopsy in a boy with severe penoscrotal hypospadias (original magnification 100×; seminiferous tubule hyalinization shown by arrowheads). (D) Ovarian dysgenesis in a patient with 46,XX primary ovarian insufficiency. Panels A–C reproduced with permission from Lin et al. (2007) (copyright: The Endocrine Society 2007). Panel D reproduced with permission from Lourenco et al. (2009) (copyright: © 2009 Massachusetts Medical Society).

Since these initial reports, an increasing number of heterozygous changes in *NR5A1* have been identified in patients with 46,XY DSD phenotypes (Fig. 2) (Lin et al., 2007; Reuter et al., 2007; Coutant et al., 2007; Köhler et al., 2008; Lourenco et al., 2009; Tajima et al., 2009; Philibert et al., 2010; Warman et al., 2010). Many cases described to date display a similar phenotype of ambiguous genitalia (or “clitoral enlargement”) at birth, a urogenital sinus, small inguinal testes and absent or rudimentary Müllerian structures. Many of these patients have intact or rudimentary Wolffian structures. Often there is biochemical evidence of partial gonadal dysgenesis and significantly impaired androgen synthesis as shown by low levels of testosterone, inhibin B and AMH, and an elevation of FSH. In some cases, testosterone levels may be relatively normal at birth, thereby resulting in a phenotype similar to partial androgen insensitivity syndrome. Gonadal histology in these cases is variable but often shows relatively preserved architecture of the testis in infancy, but reduced size of seminiferous tubules (Fig. 3A and B) (Lin et al., 2007). Usually Leydig and Sertoli cells are seen and normal or reduced numbers of germ cells are present (Fig. 3B). Although data are still limited, it is possible that the testis may be at risk of progressive dysgenetic changes, especially if it remains in an inguinal or abdominal position (Fig. 3C).

SF-1/*NR5A1* changes associated with these forms of 46,XY DSD are usually frameshift or nonsense changes, or missense changes that affect DNA-binding and gene transcription (Fig. 2). In several cohort studies, SF-1 changes have been reported in approximately 10–15% of individuals with this phenotype (Lin et al., 2007; Köhler et al., 2008). Although many of the heterozygous changes are *de novo* (or even possibly mosaic) events, about one-third of these changes have been shown to be inherited from the mother in a sex-limited dominant manner (Köhler and Achermann, 2010). These women are at potential risk of primary ovarian insufficiency (see Section 5) but, if this does not happen – or if they complete their families before the onset of early menopause – they can pass heterozygous changes in *NR5A1* on to several affected 46,XY children. This mode of transmission can mimic X-linked inheritance (Coutant et al., 2007; Lin et al., 2007; Warman et al., 2010). The features in different affected family members can be variable. In addition, one family has been reported with a homozygous missense mutation (p.D293N) in the LBD of SF-1 (Lourenco et al., 2009). This change showed partial loss-of-function (50%) in gene transcription assays and, as expected, was inherited in an autosomal recessive manner. Thus, inheritance patterns associated with *NR5A1* changes can be *de novo* (autosomal dominant), sex-limited dominant or autosomal recessive.

Further evidence for the effects of haploinsufficiency of *NR5A1* has been obtained from individuals with deletion of the locus containing *NR5A1* on one allele. In one 46,XY individual with clitoromegaly, a shallow vaginal entrance and one small palpable gonad, a 9q33 deletion including *NR5A1* was found by array comparative genomic hybridization (CGH) (van Silfhout et al., 2009). Furthermore, a contiguous gene deletion syndrome involving 9q33.3–q34.11 has been reported in a patient with genitopatellar syndrome, ambiguous genitalia and bilateral ovotestes (Schlaubitz et al., 2007).

### 4.2 The severe 46,XY DSD phenotype

**Fig. 4:**
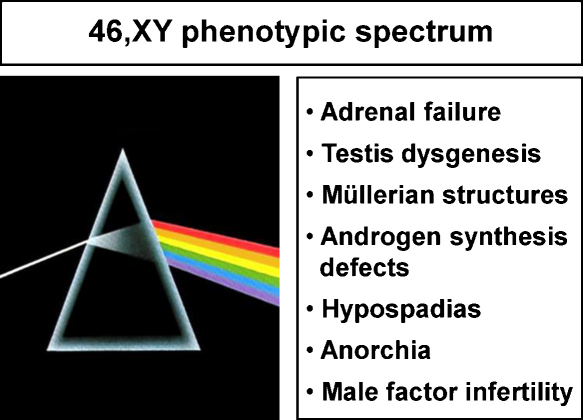
The spectrum of phenotypes that have been reported in association with SF-1/*NR5A1* changes in humans (46,XY).

At the more severe end of the spectrum, *NR5A1* mutations can be found in 46,XY girls who present in late puberty with primary amenorrhea (Fig. 4). One woman with clitoromegaly and a normal vaginal opening was found to have very small fibrous gonads in the pelvis containing disorganized tubules and Leydig cell hyperplasia (Lourenco et al., 2009). The mutation found in this case (p.M1I) affects the start codon and is likely to disrupt translation initiation of the mRNA. Other patients reported have severe gonadal dysgenesis with female external genitalia, a normal vagina, a small uterus and streak gonads. In at least two of these cases, a disruptive heterozygous change in SF-1 is present together with the p.G146A polymorphism and it has been speculated that this combination may have contributed to the more severe phenotype (Fig. 2) (Reuter et al., 2007; Köhler et al., 2008). More recently, SF-1 mutations have been described in 5/15 46,XY female adolescents presenting with primary amenorrhea and with low testosterone levels (Philibert et al., 2010). Clitoromegaly was present in three cases. A uterus was found in the one case where information was available. The authors concluded that SF-1 mutations are a frequent cause of this clinical phenotype.

### 4.3 SF-1 and hypospadias

At the less severe end of the spectrum, *NR5A1* mutations have also been detected in 46,XY patients with severe hypospadias and small inguinal testes due to partial gonadal dysgenesis and/or reduced androgen synthesis (Figs. 2 and 3C). The first case described with this phenotype harbors a heterozygous p.L437Q alteration in the ligand-like binding domain of SF-1 (Lin et al., 2007). This change is disruptive but may have partial function in some assay systems. Furthermore, three additional SF-1 alterations have been reported in a cohort of 60 boys with hypospadias (Köhler et al., 2009). Of note, these three cases were found amongst the subset of individuals with the most severe forms of hypospadias (penoscrotal). All these cases had at least one undescended testis, and reduced penile length was found in two of the three cases.

### 4.4 SF-1 and bilateral anorchia

*NR5A1* was considered a candidate gene for bilateral anorchia based on the knowledge that a subset of these cases are familial, approximately half of all cases are associated with reduced penile length, and that some genes involved in testis development may be required to maintain testicular integrity in the postnatal period. In a study of a cohort of 24 boys with bilateral anorchia (vanishing testis syndrome) in France, one individual was found to carry a heterozygous SF-1 mutation (p.V355M) (Philibert et al., 2007). One very small testis and one absent testis were found in early infancy with undetectable AMH. Testis atrophy and fibrosis was documented later in childhood. However, the patient's twin brother who harbored the same mutation is reported to have undergone normal puberty. This finding indicates that SF-1 may also play a role in maintaining the testis, but most likely the contribution of other unknown factors or altered expression of the mutant allele is responsible for the variability of phenotypes seen.

### 4.5 SF-1 and male factor infertility

Recently, Bashamboo and co-workers investigated whether changes in SF-1 could be found in a cohort of 315 men with non-obstructive male factor infertility where the underlying cause was unknown (Bashamboo et al., 2010). Men had been pre-screened for Y chromosome microdeletions and Klinefelter syndrome, and any cases where there was a potential known environmental or medical cause of infertility were excluded. Of note, there was no history of hypospadias or undescended testes. Analysis of *NR5A1* in this cohort identified changes in seven individuals. All these changes were located within the hinge region of the protein (Fig. 2). Importantly, no rare allelic variants (other than the known p.G146A polymorphism) were found following direct sequencing of the entire *NR5A1* gene in more than 600 known fertile control men, and the changes found in association with infertility were not found to be present in more than 4000 control alleles. The men who harbored *NR5A1* changes had more severe forms of infertility (azoospermia, severe oligozoospermia) and in several cases low testosterone and elevated gonadotropins were found. In the one case studied, significant fibrosis of the testis with reduced seminiferous tubules and isolated rare germ cells was seen. A serial decrease in sperm count was found in one patient studied, raising the possibility that heterozygous changes in *NR5A1* might be transmitted to offspring in some cases, especially if fatherhood occurs in young adulthood rather than later in life. Indeed, the p.G123A + p.P129L variant has been found in several patients from Central, West and North Africa, suggesting that it may be a founder effect with low level penetrance through these populations. Taken together, this study shows that changes in SF-1 may be found in a small subset of phenotypically normal men with non-obstructive male factor infertility where the cause is currently unknown. These individuals may be at risk of low testosterone in adult life and may represent part of the adult testicular dysgenesis syndrome. Making the specific diagnosis in this group of patients could be important for long-term follow-up. Furthermore, an interaction between environmental or epigenetic factors and these specific SF-1 changes may be important in regulating the phenotype. Further studies will be needed to establish whether SF-1 changes can be found in other cohorts of infertile patients.

## 5 SF-1 and ovarian insufficiency

*NR5A1* mutations have also now been identified in familial and sporadic forms of 46,XX primary ovarian insufficiency (POI) (Fig. 2) (Lourenco et al., 2009). These 46,XX patients with *NR5A1* mutations presented with either primary or secondary amenorrhea, and with a variable age of onset of features. Primary gonadal failure was shown by elevated LH and FSH levels and low estradiol. In the one case studied, an ovarian biopsy showed extensive fibrosis with no evidence of follicles (Fig. 3D). Most of these women harbored heterozygous alterations in *NR5A1* and had been identified on account of a history of 46,XY DSD and 46,XX POI in different individuals within the same family. In one large kindred a partial loss-of-function SF-1 change (p.D293N) was inherited in an autosomal recessive manner. Heterozygous SF-1/*NR5A1* changes were also found in two girls with sporadic forms of POI and no family history. Therefore, although some 46,XX women with *NR5A1* mutations have been described to have normal ovarian function and can transmit the mutation in a sex-limited dominant fashion, the detection of *NR5A1* alterations in 46,XX ovarian failure shows that SF-1 is also a key factor in ovarian development and function in humans. Disruption of SF-1 may affect the ovary at multiple levels, including reduced germ cell number, impaired stromal integrity, abnormal folliculogenesis and defective steroidogenesis. Some of these women may go through a stage of decreased ovarian reserve (with decreasing AMH and elevating gonadotropins) before manifesting clinical signs or symptoms of ovarian failure (Warman et al., 2010). Although more studies are needed to understand the natural history of these changes in more detail, and to establish how prevalent or predictable ovarian dysfunction might be, these cases do highlight that SF-1 plays a role in human ovarian function too.

## 6 The p.G146A polymorphism

A p.G146A polymorphism (rs1110061; c.624G>C) in SF-1 has been reported to be associated with micropenis or cryptorchidism in two relatively small studies of patients from Japan (Wada et al., 2005, 2006). This variant has potentially reduced function in some assay systems. Limited data have suggested that the presence of this p.G146A change together with haploinsufficiency of *NR5A1* may be associated with a more severe 46,XY DSD (see Section 4.2) (Köhler et al., 2008). However, more studies are needed before the true significance of this potential effect is known.

## 7 Overactivity of SF-1

Whilst most studies have focused on loss of function of SF-1 and human disease, it is also becoming apparent that overexpression or overactivity of SF-1 might have an important clinical effect. SF-1 *overexpression* could result from (1) genomic duplications of the chromosomal locus containing *NR5A1* resulting in biologically significant copy number variation (CNV), or from (2) upregulation of *NR5A1* gene transcription due to increased promoter/enhancer activity or following decreased promoter methylation. Alternatively, *overactivity* of SF-1 could result from (1) increased protein stabilization or reduced degradation, (2) loss of SUMOylation-dependent repression of transcriptional activity, or (3) specific changes in the structure of SF-1 that result in increased basal activity or increased affinity for native and/or alternative ligands. Several of these mechanisms are now being seen as potential causes or modifiers of human disease, which has translational implications as pharmacomodulation of SF-1 might have a potential role in the treatment of these conditions (Schimmer and White, 2010).

### 7.1 Adrenal tumorigenesis

Somatic duplications of 9q33 including *NR5A1* were originally described in 2005 in a cohort of children from Brazil with adrenocortical tumors (ACTs) (Figueiredo et al., 2005). These changes occurred largely on the background of loss of heterozygosity for the tumor suppressor gene p53 (TP53). Increased *NR5A1* expression was subsequently confirmed in an independent study of ACTs, with a higher number of pediatric tumors showing *NR5A1* overexpression compared to adult tumors (Almeida et al., 2010). Interestingly, increased nuclear SF-1 protein expression was seen in many cases, sometimes independently of detectable *NR5A1* gene expression. This finding as been supported by recent data from analysis of a large cohort of adult ACTs, which has shown a correlation between higher SF-1 protein levels and worse prognosis (Sbiera et al., 2010). Of note, SF-1 overexpression has been shown to increase proliferation and to decrease apoptosis of human adrenocortical cells, and to induce ACTs in transgenic mice (Doghman et al., 2007). SF-1 inverse agonists have been shown to inhibit adrenocortical carcinoma cell proliferation *in vitro* (Doghman et al., 2009).

### 7.2 Polycystic ovary syndrome

A heterozygous point mutation (p.R365P) has been reported in a woman with polycystic ovary syndrome (Calvo et al., 2001). The functional or clinical significance of this finding is unclear, but raises the possibility that conformational changes in the ligand-binding domain of SF-1 could have a biological effect.

### 7.3 Endometriosis

SF-1 has been shown to be expressed in endometriotic cells whereas it is not usually detected in normal endometrium (Xue et al., 2007). Part of this aberrant expression may be the result of hypomethylation of a CpG-rich region in its proximal promoter region with subsequent activation by upstream stimulatory factor 2 (USF2) (Xue et al., 2007; Utsunomiya et al., 2008). Alternatively, SF-1 activity in endometriotic tissue may be increased following stimulation of the GPR30 estrogen receptor (Lin et al., 2009). Increased SF-1 expression or activity in endometriotic tissue could result in increased activity of StAR and aromatase, resulting in increased local estrogen synthesis, a key pathological feature of this condition (Utsunomiya et al., 2008).

## 8 Conclusions

SF-1 is clearly an important mediator of adrenal and reproductive function in humans. Changes in SF-1 activity have now been reported in association with a range of human conditions. Some of these conditions are rare, sporadic events due to “private” *de novo* changes in the coding sequence of *NR5A1* in an individual's genome. However, it is also emerging that specific changes in SF-1 may contribute to more common conditions such as male factor infertility or primary ovarian insufficiency. The exact pathogenic mechanisms in some of these cases are not entirely clear and phenotypes can be variable in some cases. Therefore, changes in SF-1 are likely to predispose an individual to a given phenotype but the ultimate clinical picture may be influenced by a number of oligogenic modulators, developmental switches, epigenetic influences, environmental stimuli and even imbalanced cis-regulation of mutant versus wild-type alleles when mutations are present in a heterozygous state. More systematic studies of SF-1 in different patient cohorts will be needed to address the natural history of some of these conditions and whether detecting a change in SF-1 might predispose to late-onset adrenal insufficiency, differences in tumor risk, or be important when exploring options for fertility preservation. Furthermore, the effects of SF-1 in tumorigenesis and in other physiological systems (e.g. appetite regulation, obesity, anxiety) might be important if data from mouse studies are extrapolated into humans (Schimmer and White, 2010). The next decade of human SF-1 research promises to be as exciting as the last.

## Conflict of interest

The authors have no conflict of interest to declare.

## Role of the funding source

The authors were supported by a Wellcome Trust Senior Research Fellowship in Clinical Science (079666, to JCA) and a PhD studentship from Coordenacao de Aperfeicoamento de Pessoal de Nivel Superior (Capes, Brazil) (4798066, to BFdS). The funders played no role in the preparation of the manuscript or the decision to submit the paper for publication.

## References

[bib0005] Achermann J.C., Ito M., Ito M., Hindmarsh P.C., Jameson J.L. (1999). A mutation in the gene encoding steroidogenic factor-1 causes XY sex reversal and adrenal failure in humans. Nat. Genet..

[bib0010] Achermann J.C., Ozisik G., Ito M., Orun U.A., Harmanci K., Gurakan B., Jameson J.L. (2002). Gonadal determination and adrenal development are regulated by the orphan nuclear receptor steroidogenic factor-1, in a dose-dependent manner. J. Clin. Endocrinol. Metab..

[bib0015] Almeida M.Q., Soares I.C., Ribeiro T.C., Fragoso M.C., Marins L.V., Wakamatsu A., Ressio R.A., Nishi M.Y., Jorge A.A., Lerario A.M., Alves V.A., Mendonca B.B., Latronico A.C. (2010). Steroidogenic factor-1 overexpression and gene amplification are more frequent in adrenocortical tumors from children than from adults. J. Clin. Endocrinol. Metab..

[bib0020] Bashamboo A., Ferraz-de-Souza B., Lourenco D., Lin L., Sebire N.J., Montjean D., Bignon-Topalovic J., Mandelbaum J., Siffroi J.P., Christin-Maitre S., Radhakrishna U., Rouba H., Ravel C., Seeler J., Achermann J.C., McElreavey K. (2010). Human male infertility associated with mutations in NR5A1 encoding steroidogenic factor 1. Am. J. Hum. Genet..

[bib0025] Biason-Lauber A., Schoenle E.J. (2000). Apparently normal ovarian differentiation in a prepubertal girl with transcriptionally inactive steroidogenic factor 1 (NR5A1/SF-1) and adrenocortical insufficiency. Am. J. Hum. Genet..

[bib0030] Bland M.L., Jamieson C.A., Akana S.F., Bornstein S.R., Eisenhofer G., Dallman M.F., Ingraham H.A. (2000). Haploinsufficiency of steroidogenic factor-1 in mice disrupts adrenal development leading to an impaired stress response. Proc. Natl. Acad. Sci. U. S. A..

[bib0035] Bland M.L., Fowkes R.C., Ingraham H.A. (2004). Differential requirement for steroidogenic factor-1 gene dosage in adrenal development versus endocrine function. Mol. Endocrinol..

[bib0040] Calvo R.M., Asuncion M., Telleria D., Sancho J., San Millan J.L., Escobar-Morreale H.F. (2001). Screening for mutations in the steroidogenic acute regulatory protein and steroidogenic factor-1 genes, and in CYP11A and dosage-sensitive sex reversal-adrenal hypoplasia gene on the X chromosome, gene-1 (DAX-1), in hyperandrogenic hirsute women. J. Clin. Endocrinol. Metab..

[bib0045] Correa R.V., Domenice S., Bingham N.C., Billerbeck A.E., Rainey W.E., Parker K.L., Mendonca B.B. (2004). A microdeletion in the ligand binding domain of human steroidogenic factor 1 causes XY sex reversal without adrenal insufficiency. J. Clin. Endocrinol. Metab..

[bib0050] Coutant R., Mallet D., Lahlou N., Bouhours-Nouet N., Guichet A., Coupris L., Croue A., Morel Y. (2007). Heterozygous mutation of steroidogenic factor-1 in 46, XY subjects may mimic partial androgen insensitivity syndrome. J. Clin. Endocrinol. Metab..

[bib0055] Doghman M., Karpova T., Rodrigues G.A., Arhatte M., De Moura J., Cavalli L.R., Virolle V., Barbry P., Zambetti G.P., Figueiredo B.C., Heckert L.L., Lalli E. (2007). Increased steroidogenic factor-1 dosage triggers adrenocortical cell proliferation and cancer. Mol. Endocrinol..

[bib0060] Doghman M., Cazareth J., Douguet D., Madoux F., Hodder P., Lalli E. (2009). Inhibition of adrenocortical carcinoma cell proliferation by steroidogenic factor-1 inverse agonists. J. Clin. Endocrinol. Metab..

[bib0065] Ferraz-de-Souza B., Hudson-Davies R.E., Lin L., Achermann J.C. (2009). Novel targets of steroidogenic factor-1 (SF-1, NR5A1, Ad4BP) in the adrenal. Hormone Res..

[bib0070] Figueiredo B.C., Cavalli L.R., Pianovski M.A., Lalli E., Sandrini R., Ribeiro R.C., Zambetti G., DeLacerda L., Rodrigues G.A., Haddad B.R. (2005). Amplification of the steroidogenic factor 1 gene in childhood adrenocortical tumors. J. Clin. Endocrinol. Metab..

[bib0075] Hasegawa T., Fukami M., Sato N., Katsumata N., Sasaki G., Fukutani K., Morohashi K., Ogata T. (2004). Testicular dysgenesis without adrenal insufficiency in a 46, XY patient with a heterozygous inactive mutation of steroidogenic factor-1. J. Clin. Endocrinol. Metab..

[bib0080] Hoivik E.A., Lewis A.E., Aumo L., Bakke M. (2010). Molecular aspects of steroidogenic factor 1 (SF-1). Mol. Cell. Endocrinol..

[bib0085] Ito M., Achermann J.C., Jameson J.L. (2000). A naturally occurring steroidogenic factor-1 mutation exhibits differential binding and activation of target genes. J. Biol. Chem..

[bib0090] Köhler B., Lin L., Ferraz-de-Souza B., Wieacker P., Heidemann P., Schröder V., Biebermann H., Schnabel D., Grüters A., Achermann J.C. (2008). Five novel mutations in steroidogenic factor 1 (SF1, NR5A1) in 46, XY patients with severe underandrogenization but without adrenal insufficiency. Hum. Mutat..

[bib0095] Köhler B., Lin L., Mazen I., Cetindag C., Biebermann H., Akkurt I., Rossi R., Hiort O., Grüters A., Achermann J.C. (2009). The spectrum of phenotypes associated with mutations in steroidogenic factor 1 (SF-1, NR5A1, Ad4BP) includes severe penoscrotal hypospadias in 46, XY males without adrenal insufficiency. Eur. J. Endocrinol..

[bib0100] Köhler B., Achermann J.C. (2010). Update – steroidogenic factor 1 (SF-1, NR5A1). Minerva Endocrinol..

[bib0105] Lala D.S., Rice D.A., Parker K.L. (1992). Steroidogenic factor 1, a key regulator of steroidogenic enzyme expression, is the mouse homolog of fushi tarazu-factor I. Mol. Endocrinol..

[bib0110] Lin B.C., Suzawa M., Blind R.D., Tobias S.C., Bulun S.E., Scanlan T.S., Ingraham H.A. (2009). Stimulating the GPR130 estrogen receptor with a novel tamoxifen analogue activates SF-1 and promotes endometrial cell proliferation. Cancer Res..

[bib0115] Lin L., Gu W.X., Ozisik G., To W.S., Owen C.J., Jameson J.L., Achermann J.C. (2006). Analysis of DAX1 (NR0B1) and steroidogenic factor-1 (NR5A1) in children and adults with primary adrenal failure: ten years’ experience. J. Clin. Endocrinol. Metab..

[bib0120] Lin L., Philibert P., Ferraz-de-Souza B., Kelberman D., Homfray T., Albanese A., Molini V., Sebire N.J., Einaudi S., Conway G.S., Hughes I.A., Jameson J.L., Sultan C., Dattani M.T., Achermann J.C. (2007). Heterozygous missense mutations in steroidogenic factor 1 (SF1/Ad4BP, NR5A1) are associated with 46, XY disorders of sex development with normal adrenal function. J. Clin. Endocrinol. Metab..

[bib0125] Lin L., Achermann J.C. (2008). Steroidogenic factor-1 (SF-1, Ad4BP, NR5A1) and disorders of testis development. Sex Dev..

[bib0130] Lourenco D., Brauner R., Lin L., De Perdigo A., Weryha G., Muresan M., Boudjenah R., Guerra-Junior G., Maciel-Guerra A.T., Achermann J.C., McElreavey K., Bashamboo A. (2009). Mutations in NR5A1 associated with ovarian insufficiency. N. Engl. J. Med..

[bib0135] Luo X., Ikeda Y., Parker K.L. (1994). A cell-specific nuclear receptor is essential for adrenal and gonadal development and sexual differentiation. Cell.

[bib0140] Majdic G., Young M., Gomez-Sanchez E., Anderson P., Szczepaniak L.S., Dobbins R.L., McGarry J.D., Parker K.L. (2002). Knockout mice lacking steroidogenic factor 1 are a novel genetic model of hypothalamic obesity. Endocrinology.

[bib0145] Mallet D., Bretones P., Michel-Calemard L., Dijoud F., David M., Morel Y. (2004). Gonadal dysgenesis without adrenal insufficiency in a 46, XY patient heterozygous for the nonsense C16X mutation: a case of SF1 haploinsufficiency. J. Clin. Endocrinol. Metab..

[bib0150] Morohashi K., Honda S., Inomata Y., Handa H., Omura T. (1992). A common trans-acting factor, Ad4-binding protein, to the promoters of steroidogenic P-450s. J. Biol. Chem..

[bib0155] Parker K.L., Schimmer B.P. (1997). Steroidogenic factor 1: a key determinant of endocrine development and function. Endocr. Rev..

[bib0160] Philibert P., Zenaty D., Lin L., Soskin S., Audran F., Leger J., Achermann J.C., Sultan C. (2007). Mutational analysis of steroidogenic factor 1 (NR5a1) in 24 boys with bilateral anorchia: a French collaborative study. Hum. Reprod..

[bib0165] Philibert P., Leprieur E., Zenaty D., Thibaud E., Polak M., Frances A.M., Lespinasse J., Raingeard I., Servant N., Audran F., Paris F., Sultan C. (2010). Steroidogenic factor-1 (SF-1) gene mutation as a frequent cause of primary amenorrhea in 46, XY female adolescents with low testosterone concentration. Reprod. Biol. Endocrinol..

[bib0170] Reuter A.L., Goji K., Bingham N.C., Matsuo M., Parker K.L. (2007). A novel mutation in the accessory DNA-binding domain of human steroidogenic factor 1 causes XY gonadal dysgenesis without adrenal insufficiency. Eur. J. Endocrinol..

[bib0175] Rice D.A., Mouw A.R., Bogerd A.M., Parker K.L. (1991). A shared promoter element regulates the expression of three steroidogenic enzymes. Mol. Endocrinol..

[bib0180] Sadovsky Y., Crawford P.A., Woodson K.G., Polish J.A., Clements M.A., Tourtellotte L.M., Simburger K., Milbrandt J. (1995). Mice deficient in the orphan receptor steroidogenic factor 1 lack adrenal glands and gonads but express P450 side-chain-cleavage enzyme in the placenta and have normal embryonic serum levels of corticosteroids. Proc. Natl. Acad. Sci. U. S. A..

[bib0185] Sbiera S., Schmull S., Assie G., Voelker H.U., Kraus L., Beyer M., Ragazzon B., Beuschlein F., Willenberg H.S., Hahner S., Saeger W., Bertherat J., Allolio B., Fassnacht M. (2010). High diagnostic and prognostic value of steroidogenic factor-1 expression in adrenal tumors. J. Clin. Endocrinol. Metab..

[bib0190] Schimmer B.P., White P.C. (2010). Minireview: steroidogenic factor 1: its roles in differentiation, development, and disease. Mol. Endocrinol..

[bib0195] Schlaubitz S., Yatsenko S.A., Smith L.D., Keller K.L., Vissers L.E., Scott D.A., Cai W.W., Reardon W., Abdul-Rahman O.A., Lammer E.J., Lifchez C.A., Magenis E., Veltman J.A., Stankiewicz P., Zabel B.U., Lee B. (2007). Ovotestes and XY sex reversal in a female with an interstitial 9q33.3-q34.1 deletion encompassing NR5A1 and LMX1B causing features of Genitopatellar syndrome. Am. J. Med. Genet. A.

[bib0200] Shinoda K., Lei H., Yoshii H., Nomura M., Nagano M., Shiba H., Sasaki H., Osawa Y., Ninomiya Y., Niwa O., Morohashi K., Li E. (1995). Developmental defects of the ventromedial hypothalamic nucleus and pituitary gonadotroph in the Ftz-F1 disrupted mice. Dev. Dyn..

[bib0205] Tajima T., Fujiwara F., Fujieda K. (2009). A novel heterozygous mutation of steroidogenic factor-1 (SF-1/Ad4BP) gene (NR5A1) in a 46, XY disorders of sex development (DSD) patient without adrenal failure. Endocr. J..

[bib0210] Tremblay J.J., Viger R.S. (2003). A mutated form of steroidogenic factor 1 (SF-1 G35E) that causes sex reversal in humans fails to synergize with transcription factor GATA-4. J. Biol. Chem..

[bib0215] Umesono K., Evans R.M. (1989). Determinants of target gene specificity for steroid/thyroid hormone receptors. Cell.

[bib0220] Utsunomiya H., Cheng Y.H., Lin Z., Reierstad S., Yin P., Attar E., Xue Q., Imir G., Thung S., Trukhacheva E., Suzuki T., Sasano H., Kim J.J., Yaegashi N., Bulun S.E. (2008). Upstream stimulatory factor-2 regulates steroidogenic factor-1 expression in endometriosis. Mol. Endocrinol..

[bib0225] van Silfhout A., Boot A.M., Dijkhuizen T., Hoek A., Nijman R., Sikkema-Raddatz B., van Ravenswaaij-Arts C.M. (2009). A unique 970 kb microdeletion in 9q33.3, including the NR5A1 gene in a 46,XY female. Eur. J. Med. Genet..

[bib0230] Wada Y., Okada M., Hasegawa T., Ogata T. (2005). Association of severe micropenis with Gly146Ala polymorphism in the gene for steroidogenic factor-1. Endocr. J..

[bib0235] Wada Y., Okada M., Fukami M., Sasagawa I., Ogata T. (2006). Association of cryptorchidism with Gly146Ala polymorphism in the gene for steroidogenic factor-1. Fertil. Steril..

[bib0240] Warman D.M., Costanzo M., Marino R., Berensztein E., Galeano J., Ramirez P.C., Saraco N., Baquedano M.S., Ciaccio M., Guercio G., Chaler E., Maceiras M., Lazzatti J.M., Bailez M., Rivarola M.A., Belgorosky A. (2010). Three new SF-1 (NR5A1) gene mutations in two unrelated families with multiple affected members: within-family variability in 46,XY subjects and low ovarian reserve in fertile 46,XX subjects. Horm. Res. Paediatr..

[bib0245] Xue Q., Lin Z., Yin P., Milad M.P., Cheng Y.H., Confino E., Reierstad S., Bulun S.E. (2007). Transcriptional activation of steroidogenic factor-1 by hypomethylation of the 5′ CpG island in endometriosis. J. Clin. Endocrinol. Metab..

